# Is eGFR Slope a Novel Predictor of Chronic Complications of Type 2 Diabetes Mellitus? A Systematic Review and Meta-Analysis

**DOI:** 10.1155/2024/8859678

**Published:** 2024-01-17

**Authors:** Giovanni Sartore, Eugenio Ragazzi, Elena Deppieri, Annunziata Lapolla

**Affiliations:** ^1^Department of Medicine-DIMED, University of Padua, Padova, Italy; ^2^University of Padua, Padova, Italy

## Abstract

**Background:**

Diabetic kidney disease affects approximately 40% of patients with type 2 diabetes mellitus (T2DM) and is associated with an increased risk of end-stage kidney disease (ESKD) and cardiovascular (CV) events, as well as increased mortality. Among the indicators of decline in renal function, the eGFR slope is acquiring an increasing clinical interest. The aim of this study was to evaluate, through a systematic review of the literature and meta-analysis of the collected data, the association between the decline of the eGFR slope, chronic complications, and mortality of T2DM patients, in order to understand whether or not the eGFR slope can be defined as a predictive indicator of complications in T2DM.

**Methods:**

The review and meta-analysis were conducted according to PRISMA guidelines considering published studies on patients with T2DM. A scientific literature search was carried out on PubMed from January 2003 to April 2023 with subsequent selection of scientific papers according to the inclusion criteria.

**Results:**

Fifteen studies were selected for meta-analysis. Risk analysis as hazard ratio (HR) indicated a significant association between all events considered (all-cause mortality, CV events, ESKD, and microvascular events) for patients with steeper eGFR slope decline than subjects with stable eGFR. Calculated HRs (with 95% CI) were as follows: for all-cause mortality, 2.31 (1.70-3.15); for CV events, 1.73 (1.43-2.08); for ESKD, 1.54 (1.45-1.64); and for microvascular events, 2.07 (1.57-2.73). Overall HR was 1.82 (1.72-1.92).

**Conclusions:**

An association between rapid eGFR decline and chronic diabetes complications was demonstrated, suggesting that eGFR slope variability significantly impacts the course of T2DM and that eGFR slope should be considered as a predictor for chronic complications in patients with T2DM. According to the obtained results, the therapeutic management of the patient with diabetes should not focus exclusively on glycaemic control, and particular attention should be paid to preserve renal function.

## 1. Introduction

Approximately half of the patients affected by type 2 diabetes mellitus (T2DM) present a microvascular complication (retinopathy, neuropathy, or diabetic nephropathy), and 27% have a macrovascular complication (cardiovascular complications) [1], with a significant increase of risk than in the general population [2], leading to high healthcare costs [1–3]. Among microvascular complications of T2DM [4], diabetic nephropathy (diabetic kidney disease (DKD) is the leading cause of end-stage kidney disease (ESKD) in the Western world and is defined as the presence of impaired renal function in diabetic patients in the absence of other causes of chronic kidney disease [5, 6]. The risk of DKD development and progression depends mainly on the duration of diabetes and hypertension [7]. Approximately 30% of the patients develop microalbuminuria within 10 years of being diagnosed with diabetes, and approximately 5% progress to overt nephropathy each year [8]. With the progression of DKD, also mortality from all causes increases, and this is particularly true for cardiovascular disease (CVD): the annual mortality rate for CVD, in the absence of DKD, is 0.7%, while it rises to 2% for patients with microalbuminuria and to 3.5% for those with macroalbuminuria [8]. In order to determine the presence of DKD, the American Diabetes Association recommends the measurement of spot urinary albuminuria through the urinary albumin/creatinine ratio (UACR) and the evaluation of the estimated glomerular filtration rate (eGFR). If albuminuria is high, the data must be confirmed by repeating the UACR within six months [5]. The diagnosis of DKD is based on an increase in urinary albumin excretion (UACR ≥ 30 mg/g creatinine) and/or a decrease in eGFR (eGFR < 60 mL/min/1.73 m^2^) in a patient affected by diabetes [4, 5, 9].

A relevant problem with DKD is that approximately 20% of the patients progress to require dialysis without receiving treatment from a nephrologist [10, 11]. Current guidelines recommend referral to a nephrologist when the eGFR falls below 30 mL/min/1.73 m^2^, when the aetiology of the disease is unclear, and when there are management concerns (resistant hypertension, worsening of albuminuria, and rapid worsening of eGFR) [5]. Increasing attention has been paid on the role of short-term changes in eGFR as a prognostic method of diabetes-related complications, and two meta-analyses demonstrated a significant association between a decline in eGFR and the risk of ESKD and mortality [12, 13]. A strong limitation of this approach was linked to the fact that eGFR change was derived by two measurements only, ignoring the behaviour of the parameter over time. Therefore, a more complete approach on the microvascular kidney complications has recently been introduced, based on eGFR slope using multiple eGFR measurements, indicating annual change in eGFR [14], defining its positive or negative slope.

The aim of the present study was to evaluate the association between eGFR slope variability, chronic complications, and mortality in patients with T2DM. Through a systematic review of the literature and the subsequent meta-analysis of the collected data, the predictive role of the eGFR slope was investigated by comparing patients with a decline in the eGFR slope to patients with a stable eGFR slope. As far as we know, this meta-analysis is the first breakthrough on T2DM patients and is aimed at bringing together all published data in a single study. The overview of the data will hopefully be useful to extend the knowledge on the role of this new indicator specifically in the T2DM patient population.

## 2. Materials and Methods

The meta-analysis was conducted according to PRISMA Statement guidelines [15], following the suggested checklist of items (Supplementary material: PRISMA checklist (available here)). The scientific literature (English language of publication) search was performed through the PubMed database from January 2003 to April 2023 with the following keywords: [(“eGFR decline” OR “eGFR slope”) AND “type 2 diabetes mellitus” OR “microvascular complications” OR “macrovascular complications”]. Each study identified was evaluated independently by two reviewers (E.D. and G.S.) in order to assess the suitability for the meta-analysis.

The eligibility criteria were defined on the basis of the PICOS criteria [16, 17]:

- P: population: patients older than 18 years diagnosed with T2DM
- I: condition investigated: measurement of the variability of the eGFR slope evaluated by hazard ratio (HR)
- C: comparison method: logistic or Cox regression analysis for outcome risk prediction
- O: outcome: risk of macro-/microvascular complications, all causes of death
- S: type of study: any type of clinical trial (randomized controlled trial, cohort study, etc.)

All the articles that met the requirements were taken into consideration, regardless of the age of the participants (as long as they were adults). Reviews, editorials, and case reports were excluded. Comprehensive articles on potentially relevant studies were downloaded and reviewed for inclusion.

Adverse outcomes considered were all-cause mortality (ACM), cardiovascular (CV) events, end-stage kidney disease (ESKD), and microvascular complications. For cardiovascular events, major cardiovascular events (MACE) were considered as a composite criterion combining cardiovascular mortality, nonfatal myocardial infarction, and nonfatal stroke. For microvascular complications, reference was made to retinopathy and diabetic neuropathy. Diabetic nephropathy was not included within microvascular complications because it was investigated by ESKD. ESKD was defined as end-stage kidney disease with initiation of replacement therapy, either by dialysis or kidney transplantation.

Risk of bias was evaluated for the included studies according to currently suggested methods [18] and the ROBINS-E tool [19]. The risk of bias approach was used in the context of the systematic review providing an examination of the strength of evidence about the presence of potential effect of an exposure on an outcome. Each of the seven bias domains evaluated is addressed using a series of questions that aim to gather important information about the study and the analysis being assessed [19]. Supplementary Figure 1 and 2 show the results of the risk of bias assessment of the studies included in the meta-analysis.

Meta-analysis was performed using Review Manager (RevMan) (computer program) version 5.4.1 [20]. The analysis was stratified by the presence of data on eGFR slope variability using the hazard ratio (HR) available in the reviewed articles. HR values were extracted directly from the studies. In the case of multiple HR estimates based on different models, presented in a single article, all proposed data were considered and individually evaluated. This approach was selected in order not to exclude any valuable information included in the published study.

Subgroup analyses and overall values were presented as forest plots. The analysis was performed using the random effects method [21, 22].

The measure of the magnitude of variation (heterogeneity) between the effects presented in different studies was quantified by Tau^2^. Heterogeneity was also evaluated by *I*^2^ statistic based on the *χ*^2^ test [23] considering the following suggested levels of heterogeneity: 0% to 40% may not be important; 30% to 60% may represent moderate heterogeneity; 50% to 90% may represent substantial heterogeneity; and from 75% to 100%, considerable heterogeneity. Testing for the overall effect for each group and for all subgroups was performed based on the *z* distribution and the significance results provided [24].

## 3. Results

**Figure 1:**
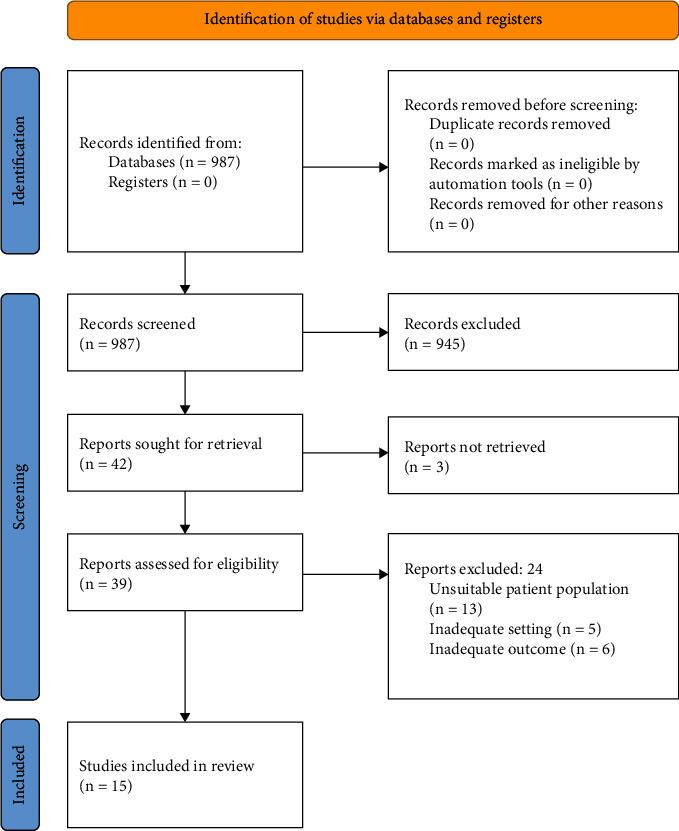
Flowchart, according to PRISMA 2020 guidelines [15], for the selection of studies used in this meta-analysis.

Figure 1 presents the results of the scientific literature search and the study selection. We identified 987 studies from PubMed, and after excluding studies that did not meet the previously described inclusion criteria, 39 studies were evaluated in detail. Of these, 24 were excluded for reasons related to the definition of adverse outcomes, the population selected in the study, and the carried-out method. We included 6 articles regarding the association between all-cause mortality (ACM) and eGFR decline (defined as eGFR slope), 8 studies regarding the association between cardiovascular events and eGFR slope decline, 11 on the association between ESKD and eGFR slope decline, and 2 studies on the association between microvascular complications and eGFR slope decline.  Table 1 shows the details of the studies related to the eGFR slope considered in the meta-analysis. Risk of bias, related to confounding, measurement of exposure, selection of participants, missing data, outcome measurement, or selection, was rated as “low risk” for the selected studies.

The cutoffs used to define patients as belonging to the declining eGFR slope and stable eGFR category differ according to the study considered, since there is no common international agreement in this regard. Therefore, the method of defining the eGFR slope has been explained for each study; moreover, the different subgroups of patients and the different risk estimation models were taken into consideration, if present.

### 3.1. eGFR Slope and All-Cause Mortality

**Figure 2:**
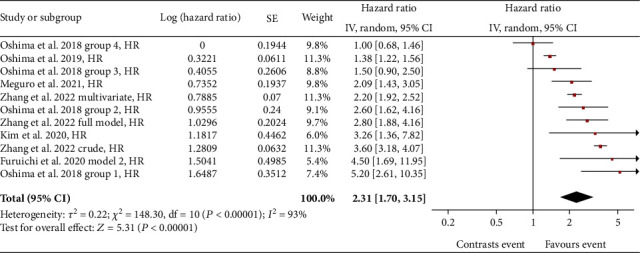
Forest plot of association between all-cause mortality (ACM) and eGFR slope decline, random effect model. Values are expressed as hazard ratio (HR), taken directly from published data. Here and in the following figures, each study is represented by a point estimate of the intervention effect, completed with a horizontal line extending either side (indicating the 95% confidence interval (95% CI)); the summary result is represented as a diamond at the bottom of each group.

A total of 6 studies were included in the analysis between eGFR slope variability and all-cause mortality (ACM) in patients with T2DM. Comparing patients with declining eGFR slope versus patients with stable eGFR slope, an overall significant risk (HR) for all-cause mortality of 2.31 (95% confidence interval (CI): 1.70-3.15) was found (Figure 2).

The total number of T2DM patients considered in the 6 studies amounted to 83,200 subjects. In the study by Oshima et al. [25], the risk of mortality, compared to patients with stable eGFR, was stratified into 4 groups based on the percentage reduction of the eGFR slope from baseline over a period of 3 years: -53% for group 1, -40% for group 2, -30% for group 3, -and 20% for group 4. An increasing mortality risk was found linked to the increase in the decline of eGFR. In Zhang et al.'s study [26], data from 5189 patients with rapidly declining eGFR slope (≤ -5 mL/min/1.73 m^2^/year) were compared with patients in whom the eGFR slope was stable (> −1 to ≤1 mL/min/1.73 m^2^/year). The result was a HR of 3.6 for the “crude model” in which only the eGFR slope category was considered; a HR of 2.20 was found for the “multivariable model” in which the eGFR slope was adjusted for age, gender, and comorbidities; finally, a HR of 2.80 was reported for the “full model,” in which an adjustment based on the eGFR was added to the multivariate model. In the study by Meguro et al. [27], patients with an eGFR slope of -18.2% per year decline from baseline were considered fast decliners. Compared with nondeclining patients, the result was a statistically significant HR of 2.09 for all causes of death. In the Kim et al. study [28], patients with eGFR slope decline were defined as having a 30% reduction from baseline eGFR. Compared with patients with stable eGFR slope, the HR was 3.26. In the study of Oshima et al. [14], patients with an annual reduction in the eGFR slope < −1.63 mL/min/1.73 m^−2^/year were significantly associated with a higher risk of mortality (HR 1.38) compared to patients with stable eGFR slope (between −1.63 and 0.33 mL/min/1.73 m^−2^/year). As regards the Furuichi et al. study [29], the data (HR and 95% CI) for the meta-analysis were estimated by a graphical approach from published plots, since the numerical values were not available. Baseline patients were defined as those whose eGFR slope was ≥0 and <5 mL/min/1.73 m^−2^/year, and patients with declining renal function were defined as those whose eGFR slope was ≤-5 mL/min/1.73 m^2^/year. After adjustment for age, sex, haemoglobin, systolic blood pressure, and albuminuria, the HR was 4.50.

### 3.2. eGFR Slope and Cardiovascular Events

**Figure 3:**
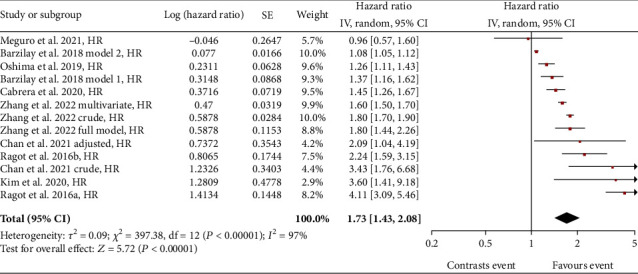
Forest plot of association between CV events and eGFR slope decline, random effect model.

A total of 8 studies were included in the analysis of eGFR slope variability versus cardiovascular (CV) events in T2DM patients. Comparing patients with declining eGFR slope and patients with stable eGFR slope revealed an overall significant risk (HR) for cardiovascular events of 1.73 (95% CI: 1.43-2.08) (Figure 3). The total number of patients affected by diabetes considered in these studies amounted to 125,817 subjects.

Details of studies by Meguro et al. [27], Oshima et al. [14], Zhang et al. [26], and Kim et al. [28] have already been described in the previous paragraph. In Barzilay et al.'s study [30], patients with eGFR slope decline were analysed according to two different models. In model 1, patients with eGFR slope decline were considered those with an eGFR slope < −5 mL/min/1.73m^2^/year. In model 2, a 5% reduction from baseline of the eGFR slope was used as a cutoff to define patients with decline. In both cases, a statistically significantly higher risk of cardiovascular events was demonstrated compared to patients with stable eGFR. In the study by Cabrera et al. [31], the decline of the eGFR slope was defined for a value <–3 mL/min/1.73 m^2^ in 2 years. In the Chan et al. study [32], the decline in eGFR slope was considered according to a cutoff < −30% from baseline with a mean follow-up period of 13.9 ± 9.1 months. The data presented in the forest plot of Figure 3 refer to the raw data (crude model) and the data adjusted for age, gender, duration of diabetes, comorbidities, HbA1c levels, eGFR, lipid profile, BMI, and antiplatelet/antihypertensive/diuretic/antihypoglycaemic therapy. In the Ragot et al. study [33], the cutoff for eGFR slope decline was defined as eGFR slope < −5 mL/min/1.73m^2^/year. The association analysis between eGFR slope variability and CV event risk was performed on two distinct cohorts of T2DM patients, which are defined in Figure 3 as “a” and “b” and refer to the SURDIAGENE and DIABHYCAR studies, respectively.

### 3.3. eGFR Slope and ESKD (End-Stage Kidney Disease)

**Figure 4:**
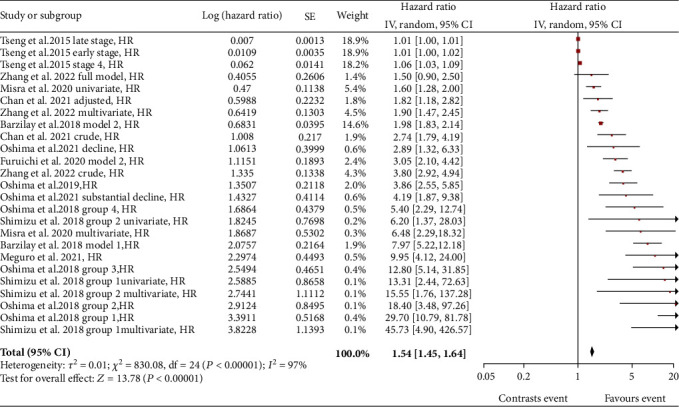
Forest plot of association between end-stage kidney disease (ESKD) and eGFR slope decline, random effect model.

Eleven studies (on a total of 169,840 subjects) were included in the analysis of eGFR slope variability linked to end-stage kidney disease (ESKD) in T2DM patients. Comparison of patients with eGFR slope decline versus patients with stable eGFR slope gave a significant overall risk (HR) for ESKD of 1.54 (95% CI: 1.45-1.64) (Figure 4).

Also for this complication, different subgroups of patients with different risk estimation models were taken into consideration, if present. In particular, the studies by Meguro et al. [27], Oshima et al. [14, 25], Zhang et al. [26], Kim et al. [28], Barzilay et al. [30], and Chan et al. [32] have already been described in above sections. In the study by Tseng et al. [34], nephropathic T2DM patients were divided, based on the disease stage, into early stage 3, late stage 3, and stage 4. The cutoff to define the decline of the eGFR slope was chosen as -10 mL/min/1.73 m^2^/year. For each of the three groups, the risk of developing ESKD increased up to 6.7% for stage 4 patients, in patients with a rapidly declining eGFR slope compared with patients with a stable eGFR slope. In the study by Oshima et al. [35], T2DM patients were divided into two categories based on the decline of the eGFR slope. Figure 4 refers to the “decline” group, indicating patients with a decline in the eGFR slope by 2 and 5 mL/min/1.73m^2^/year, and to the “substantial decline” group, considering patients with an eGFR slope < −5 mL/min/1.73m^2^/year. The risk of progression to ESKD was significantly higher in the “substantial decline” group.

In the study by Misra et al. [36], eGFR slope <–5 mL/min/1.73 m^2^/year was chosen for the decline of the eGFR slope. Univariate and multivariate analyses including albumin/creatinuria ratio and HbA1c level were performed. Both analyses defined an association between the decline in the eGFR slope and progression of renal disease to end stage. In the study by Shimizu et al. [37], two classes of patients were considered: class 1 with eGFR slope decline ≤ −50% in two years and class 2 with eGFR slope decline between -50 and -30% in 2 years. For each of the groups, univariate and multivariate analyses were performed taking into consideration age, gender, HbA1c, systolic blood pressure, total cholesterol, and BMI. An increased risk is also confirmed in this study of ESKD in patients with eGFR slope decline compared to patients with stable eGFR (defined as eGFR slope -30% to 0%). In the study by Furuichi et al. [29], similar to what is described in the paragraph on all-cause mortality risk, the data were obtained by graphical extrapolation, since they were not made explicit in the study. Baseline patients are defined by eGFR slope ≥ 0 and <5 mL/min/1.73 m^2^/year and patients with renal function decline by eGFR slope ≤ −5 mL/min/1.73 m^2^/year. After adjustment for age, gender, haemoglobin, systolic blood pressure, and albuminuria, the HR was 3.05.

### 3.4. eGFR Slope and Microvascular Complications

**Figure 5:**
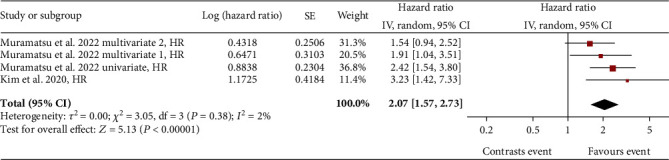
Forest plot of association between microvascular events and eGFR slope decline, random effect model.

A total of 2 studies (with overall 8048 subjects) were included in the analysis of eGFR slope variability and microvascular complications in T2DM patients. Comparing patients with declining eGFR slope versus patients with stable eGFR slope, the overall significant risk (HR) for microvascular complications was 2.07 (95% CI: 1.57-2.73) (Figure 5).

In Muramatsu et al.'s study [38], the association between eGFR slope decline of -40% from baseline and autonomic neuropathic microvascular complications was investigated according to a univariate model and two multivariate models: in the multivariate model 1, the confounding effect of nonautonomic complications was taken into consideration, while in the multivariate model 2, the independence of the association between decline in the eGFR slope and microvascular complications was studied. In the study by Kim et al. [28], the association between a 30% decline in the eGFR slope and microvascular complications was investigated and demonstrated in a population of patients with T2DM. From the meta-analysis of both studies (Figure 5), although the multivariate model 2 from Muramatsu et al. [38] did not prove a significant risk, the overall HR suggests an association between eGFR slope and microvascular complications.

### 3.5. Role of eGFR Slope in Overall Risk of Complications

**Figure 6:**
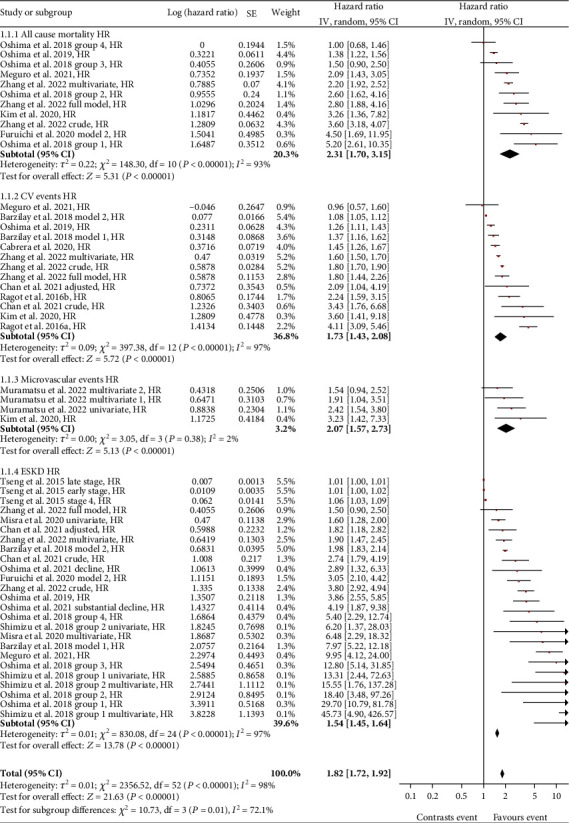
Forest plot of association between the decline of all the considered outcomes and eGFR slope, random effect model.

An overall significant risk (HR) of 1.82 (95% CI: 1.72-1.92) (Figure 6) was obtained by means of eGFR slope after combining all the considered complications. This risk measure includes all-cause mortality together with macro- and microvascular complications, giving an overview of how the decline in the eGFR slope reflects the progression of T2DM. Although the meta-analysis reveals the presence of a non-negligible heterogeneity (Tau^2^ = 0.01; *I*^2^ = 98%), the datum appears as statistically significant, as were the data taken by individual complication, and this reinforces the value of the eGFR slope parameter for the complication risk evaluation in the clinical history of T2DM patient.

## 4. Discussion

The present meta-analysis shows that for all-cause mortality (ACM), major cardiovascular events, ESKD, and microvascular events, the decline in eGFR slope represents a factor significantly associated with the clinical events of interest in T2DM patients. To our knowledge, this study is the most comprehensive evaluation on the association between eGFR slope variability and diabetes complications in patients with T2DM.

The results show a stronger correlation for ACM where the HR is 2.31, identifying a risk rate of death for patients with eGFR slope decline that is 230% that of patients with stable eGFR slope. The other adverse outcomes considered also gave a positive association with the decline of the eGFR slope, being HR of 2.07 for microvascular events, HR of 1.73 for CV events, and HR of 1.54 for ESKD.

Another interesting datum that emerged is the direct correlation between different decline rate of the eGFR slope and risk of adverse event. Considering the study by Oshima et al. [25], in which 4 classes of decline in the eGFR slope were defined, the greater is the risk for the outcome (ACM), the greater is the decline in the eGFR slope. A similar conclusion can be made by looking at the study by Shimizu et al. [37] who instead investigated the association between eGFR slope and ESKD.

If eGFR (as a single measurement) is an established predictor of ESKD, CV events, and ACM [5, 39], the role of eGFR slope in the evaluation of cardiovascular, renal, and microvascular complications both in patients with and without diabetes is less clear because it is less investigated. In this perspective, the present work is aimed to be a first step in defining the role of eGFR slope as a new risk predictor for the chronic complications of T2DM. Since this meta-analysis is the first one performed on T2DM patients, the results are comparable only with meta-analyses performed on the general population/populations with chronic kidney disease (CKD).

The data collected in previous meta-analyses are in line with the results of our study; in a meta-analysis performed on 12 CKD cohorts and 22 non-CKD cohorts (general population/with cardiovascular risk), a statistically significant association was found between the decline in the eGFR slope (<-5 mL/min/1.73 m^2^/year) and ACM [40]. This association was also observed after adjustment for the current eGFR value suggesting that the eGFR slope may provide additional information compared to the single glomerular filtration data. The reported HR for the CKD cohorts is 1.25, while for the non-CKD cohorts, it is 1.15; comparing the results with our meta-analysis (HR 2.31), it is possible to observe a closer correlation between eGFR slope decline and ACM in T2DM patients than in patients with chronic kidney disease and, even more, than in the general population [40]. It should be considered that the different risk obtained in the present meta-analysis in comparison to the reported study by Naimark et al. [40] could be affected also by the different sample size (about 380,000 patients in this meta-analysis versus >1.2 million subjects in the general population of the study by Naimark et al. [40]). Moreover, due to the design of the present analysis, the presence of multiple HR values coming from a single study may have influenced the overall estimate, which should be taken as a provisional datum, to be better focused when additional independent clinical studies will appear in the literature. The present finding needs to be further investigated, but it already indicates that eGFR slope can be considered a relevant complication risk indicator, especially in patients affected by diabetes mellitus.

Another meta-analysis investigated the role of the eGFR slope in predicting ESKD [41], demonstrating an association between eGFR slope decline (<−6 mL/min/1.73m^2^/year and < −3 mL/min/1.73 m^2^/year) and ESKD; however, this association was found as significant in the CKD cohorts only. The other cohorts (general population/population at CV risk) showed similar trends, but greater heterogeneity and lack of statistical significance indicated that the association refers mainly to patients already diagnosed with CKD. The reported HRs for the CKD cohorts were 2.28 and 1.73 for eGFR slopes < −6 and < −3 mL/min/1.73 m^2^, respectively [41]. Comparing these data with the results of our meta-analysis in T2DM (HR 1.54), a higher ESKD risk association appears in the nondiabetic CKD population, when the eGFR slope decline is particularly rapid, but a similar correlation occurs between nondiabetic CKD subjects and T2DM patients when the eGFR slope decline is less rapid. A fundamental factor to consider is that the eGFR slope seems to provide additional information compared to the single measurement of eGFR both regarding cardiovascular risk and regarding the progression of kidney disease. The present study also extends this concept to T2DM patients, whose cardiovascular and renal risks are enhanced if compared to the general population.

Various mechanisms have been proposed that may explain the importance of eGFR trends over time (eGFR slope) versus single eGFR data in predicting mortality/ESKD. First of all, the single eGFR data based on creatinine can reflect not only a change in glomerular filtration rate but also an alteration of muscle mass or malnutrition. In addition, patients with a rapid decline in eGFR in the clinical history will tend to maintain this trend resulting in lower individual eGFR levels which are associated with a higher risk of mortality/micro- and macrovascular complications [40]. On the other hand, an antecedent decline in the eGFR slope could underlie patient comorbidities and not be directly related to actual renal function. Comorbidities must therefore be considered as confounding factors to avoid erroneous conclusions [42].

This meta-analysis further strengthens the value of eGFR slope by identifying it as a parameter to be considered in the general evaluation of the patient affected by diabetes. If, as the study suggests, the trend of the eGFR slope is so strongly associated with diabetic complications, the predictive and therefore preventive role that this parameter assumes is clear.

From this point of view, the study fully fits into the emerging trend in the field of diabetic nephropathy: progressive kidney decline (the eGFR slope), and not albuminuria, is the hallmark of disease progression. In the Joslin Kidney Study conducted on patients with T2DM and normoalbuminuria, significant renal decline (of at least 3 mL/min/year) occurred in 20% of subjects, and this was associated with a higher risk of disease progression to terminal this stage (ESKD) [43]. Progressive decline in renal function has been recognized as the earliest clinical manifestation of diabetic nephropathy, and frequently, it begins in the absence of albuminuria.

In the present meta-analysis, only the hazard ratio (HR) and not the odds ratio (OR) was considered as a risk parameter. This choice was due to the fact that there were too few studies with OR as a risk parameter in the literature to combine them in a meta-analysis. This led to the exclusion of studies such as that by Jiang et al. [44] and by Cho et al. [45], despite the significance of the results on the variation of the eGFR slope. The consideration of only studies with data expressed as HR cannot be defined as a limitation of the present meta-analysis: several studies [46, 47] show that OR is a risk parameter which in this context gives less useful information than HR. The OR, if compared to HR, tends to overestimate the risk and is a static measure that does not consider the rates (variations over time), thus assessing the risk at a specific point in time. The HR, on the contrary, considers the rates and provides information on the phenomenon in progress over time, defining itself as the representation of the risk in progress.

As regards the question of the heterogeneity of HR data, the results obtained present a relevant variability: the measure of heterogeneity, expressed as the *I*^2^ statistic, has values greater than 90%. However, the distribution of data in the forest plot suggests that almost all of the studies, although characterized by sustained overall heterogeneity, are located beyond the null effect line, being associated with an overall increase in the risk of the outcome. Also in this context and as mentioned above, the presence of multiple HR estimations coming from the same study, with sometimes a wide range of variability, must be taken into account in order to explain the overall distribution of data.

The most important problem linked to eGFR slope is undoubtedly the lack of its standardization both as regards the cutoff definition of patients in rapid decline (“fast decliners”) and as regards the number of eGFR measurements, that is, the interval at which they are taken and the chosen follow-up. The 2012 KDIGO (Kidney Disease: Improving Global Outcomes) guidelines define a significant decline in the eGFR slope as a reduction in eGFR greater than −5 mL/min/1.73 m^2^/year [39]. However, several studies on T2DM patients, but also on the general population [14, 31], have used less stringent cutoffs (-3 mL/min/1.73 m^2^/year), finding equally important risks both associated with the progression of renal disease and with cardiovascular events. Using eGFR slopes smaller than -5 mL/min/1.73m^2^/year should be evaluated as more practical surrogate endpoints for people affected by diabetes.

The problem with defining the cutoffs is due, in part, to the fact that people with diabetes have a nonconstant rate of decline in eGFR and, in part, to the fact that the observation period for adverse events is not standardized, and it varies among the studies. Longer observation periods may help to distinguish the effect of small changes in eGFR slope on chronic renal dysfunction [27]. Indeed, it should be remembered that eGFR slope studies are often performed on patients starting new treatments for kidney disease such as RAAS inhibitors which cause an initial acute reduction in eGFR, although in the long term, they give a beneficial effect.

To address nonlinearity, measurement variability, and potential initial short-term effects of therapy, the Chronic Kidney Disease Epidemiology Collaboration (CKD-EPI) proposed a two-slope model to determine the eGFR slope [48]. The classic linear model uses a single slope (given by at least 2 eGFR measurements) to determine the decline of the eGFR slope. On the contrary, the two-slope model makes use of a first short-term phase (first 3 months of follow-up) and a second long-term phase. It allows to consider any effects, whether positive or negative, which occur in the short term on the eGFR and which would otherwise have an excessive impact on the eGFR slope itself. Considering the short-term effect, the long-term effect, and the total slope, this model gives a more realistic and overall picture of the behaviour of eGFR over time [48]. In general, it should be remembered that the influence of the short-term effect on total slope is greater when follow-up is short and when the rate of progression of eGFR decline is slow [48].

Considering the heterogeneity of eGFR slope measurement, in the present meta-analysis, all the studies using different follow-up periods and cutoffs were extensively and individually explained in Results, in order to evaluate, besides the overall calculated risk value, also any possible adjunctive confounding variables. Surely, it is desirable that a standardization on the eGFR slope parameter will be achieved, in order to make the comparison between studies simpler and more effective. The role of eGFR slope in the evaluation of the patient with diabetes should be highlighted, as happens with glycated haemoglobin in controlling the progress of the disease. Far more studies have been conducted on the relationship between HbA1c (glycated haemoglobin) variability and diabetic complications, than studies (such as this meta-analysis) conducted on eGFR slope variability and diabetic complications; this, together with the fact that the glycated haemoglobin measurement is standardized, differently from what happens for the eGFR slope and the difficulties described above, explains why in clinical practice, the glycaemic parameter is used almost exclusively for the evaluation of patients. However, if the data relating to HbA1c and the eGFR slope are compared, it emerges that both are particularly strong prognostic indicators and that even the eGFR slope seems to be stronger. Considering data from similar meta-analyses on HbA1c [49, 50], the HR of HbA1c variability for ACM is 1.33 versus 2.31 in this meta-analysis, and the HR for CV events is similar (1.40 for HbA1c and 1.73 for eGFR slope), the same for the progression of renal disease (1.29 for HbA1c and 1.54 for eGFR slope).

## 5. Conclusions

In conclusion, the concept of eGFR slope as a surrogate endpoint of renal function and predictor of cardiovascular and renal complications is a concept of recent acquisition and still little or not used in clinical practice. This is even more true regarding patients affected by diabetes, a condition where studies are lacking.

Based on the most recent available literature data, the results obtained from this meta-analysis suggest that the decline in glomerular filtration over time is a significant indicator for chronic complications in patients with T2DM. This assumption opens important future perspectives in the field of the care of T2DM patient, but also of the early diagnosis of diabetes complications, considering the impact that these have on patient mortality. Research in the field of the eGFR slope is desirable, in order to make this parameter usable in clinical practice, in particular regarding its standardization, allowing patients to be stratified on the basis of the rate of decline of filtration. The renewed approach to diabetes therapy focused on its complications suggests that although glycaemic control is undoubtedly relevant, it should not be the only indicator to take into consideration. Acting with drugs that preserve kidney function and therefore prevent the rapid decline of the eGFR slope is desirable and in line with the most recent recommendations.

## Figures and Tables

**Table 1:** Details of the studies related to the eGFR slope considered in the meta-analysis. Studies are presented in chronological order of publication.

Study	Type of study; time interval considered; country	Overall sample size (*n*)	Age (y) of patients (mean ± SD, or median and range)	Gender (male %)	Inclusion criteria	Methodological notes	Evaluated complications of interest for meta-analysis
Tseng et al. [34]	Longitudinal retrospective cohort study; Sept. 1999-Oct. 2000; USA	70,598	70 ± 9	97.5	Veterans Health Administration patients with diabetes in the fiscal year 2000 and CKD (stages 3–4) with up to 5 years of follow-up	Cox proportional hazards models on different CKD	ESKD
Ragot et al. [33]	Prospective monocentric cohort and clinical trial; 2002-2012; France	1,140 2,572	65 ± 11 65 ± 68	58 73	Patients with T2DM from the SURDIAGENE study (discovery cohort) and DIABHYCAR study (replication cohort)	Cox models on discovery and replication cohorts	CV events
Barzilay et al. [30]	Multicenter (513), randomized, nonblinded trial; 1994-2002; USA	6,656	66 ± 7	—	Patients with T2DM from the Antihypertensive and Lipid Lowering Treatment to Prevent Heart Attack Trial (ALLHAT)	Hazard ratios from Cox regression models adjusted for baseline eGFR and several covariates and factors	CV events and ESKD
Oshima et al. [25]	Cohort study; 1985-2010; Japan	4,814	60 ± 11	57	Patients with T2DM	Multivariable Cox proportional hazards models	ESKD and ACM
Shimizu et al. [37]	Nationwide observational study; Jul. 2009-Dec. 2015; Japan	456	66 (59-73)	65	Patients with T2DM and clinically suspected diabetic nephropathy from the Japan Diabetic Nephropathy Cohort Study (JDNCS)	Cox proportional hazards model	ESKD
Oshima et al. [14]	Randomized controlled trial and post-trial observational study; Jun. 2001–Mar. 2003; 20 countries	8,879	66 ± 6	58	Individuals with T2DM aged ≥55 years at high risk of cardiovascular events	HR from multivariate analysis using Cox proportional hazards model	ACM, CV events, ESKD
Cabrera et al. [31]	UK Clinical Practice Research Data Link GOLD (CPRD) database; 1995-2015; UK	30,222	71 ± 11	46	Newly diagnosed CKD subjects from a prevalent population of T2DM	Cox proportional hazards regression models	CV events
Furuichi et al. [29]	Multicenter retrospective study; 1985-2016; Japan	377	59	72	Patients with diagnosed diabetic nephropathy	Cox regression analysis	ACM, ESKD
Kim et al. [28]	2 × 2 factorial randomized controlled trial; 2001-2006; 200 centers in Australasia, Asia, Europe, and North America	7,217	66 ± 7	60	T2DM patients at high risk of vascular disease from the ADVANCE (Action in Diabetes and Vascular Disease: Preterax and Diamicron Modified Release Controlled Evaluation) trial	Cox proportional hazards models	ACM, CV events, microvascular complications
Misra et al. [36]	Longitudinal cohort study; 2007-2016; Canada	50	56 (47–63)	64	Adult patients affected by diabetes with biopsy-proven diabetic nephropathy	Kaplan–Meier survival curves and logrank (Mantel-Cox) analysis	ESKD
Chan et al. [32]	Retrospective study; 2016-2018; Taiwan	11,769	59 ± 12	56	Patients with T2DM treated with sodium-glucose co-transporter-2 (SGLT2is) inhibitors	Multivariate Cox proportional hazards regression	CV events, ESKD
Meguro et al. [27]	Multicenter randomized controlled trial; May 2010–Oct. 2013; Japan	4,461	63 ± 11	48	T2DM patients with hypercholesterolemia and diabetic retinopathy from EMPATHY (standard versus intEnsive statin therapy for hypercholesteroleMic Patients with diAbetic retinopathy) trial	Cox regression analysis	CV events, ESKD, ACM
Oshima et al. [35]	Multicenter observational cohort study; 1985-2010; Japan	4,328	60 ± 11	57	T2DM patients	Cox proportional regression	ESKD
Muramatsu et al. [38]	Retrospective observational cohort study; 2005-2018; Japan	831	56 ± 12	73	T2DM patients	Cox proportional hazards model	CAN
Zhang et al. [26]	Retrospective, observational cohort study; Jan. 2012-Aug. 2019; Japan	57,452	66 ± 14	52	Patients from the Japanese Medical Data Vision database	Cox proportional hazards models with multivariable adjustment	ACM, CV events, ESKD

Abbreviations: CKD: chronic kidney disease; CAN: cardiovascular autonomic neuropathy; CV events: cardiovascular events; ESKD: end-stage kidney disease; ACM: all-cause mortality.

## Data Availability

All data extracted and analysed are included within the manuscript.

## Supplementary Materials

Supplementary 1 PRISMA checklist.

Supplementary 2 Supplementary Figure 1 and 2: risk of bias assessment.
