# Supplementary material for: Is eGFR Slope a Novel Predictor of Chronic Complications of Type 2 Diabetes Mellitus? A Systematic Review and Meta-Analysis
Source: J Diabetes Res. 2024 Jan 17;2024:8859678. doi: 10.1155/2024/8859678 (PMC10807937; doi:10.1155/2024/8859678)
Supplement: Supplementary 2 — Supplementary Figure 1 and 2: risk of bias assessment. [file 8859678.f2.docx]

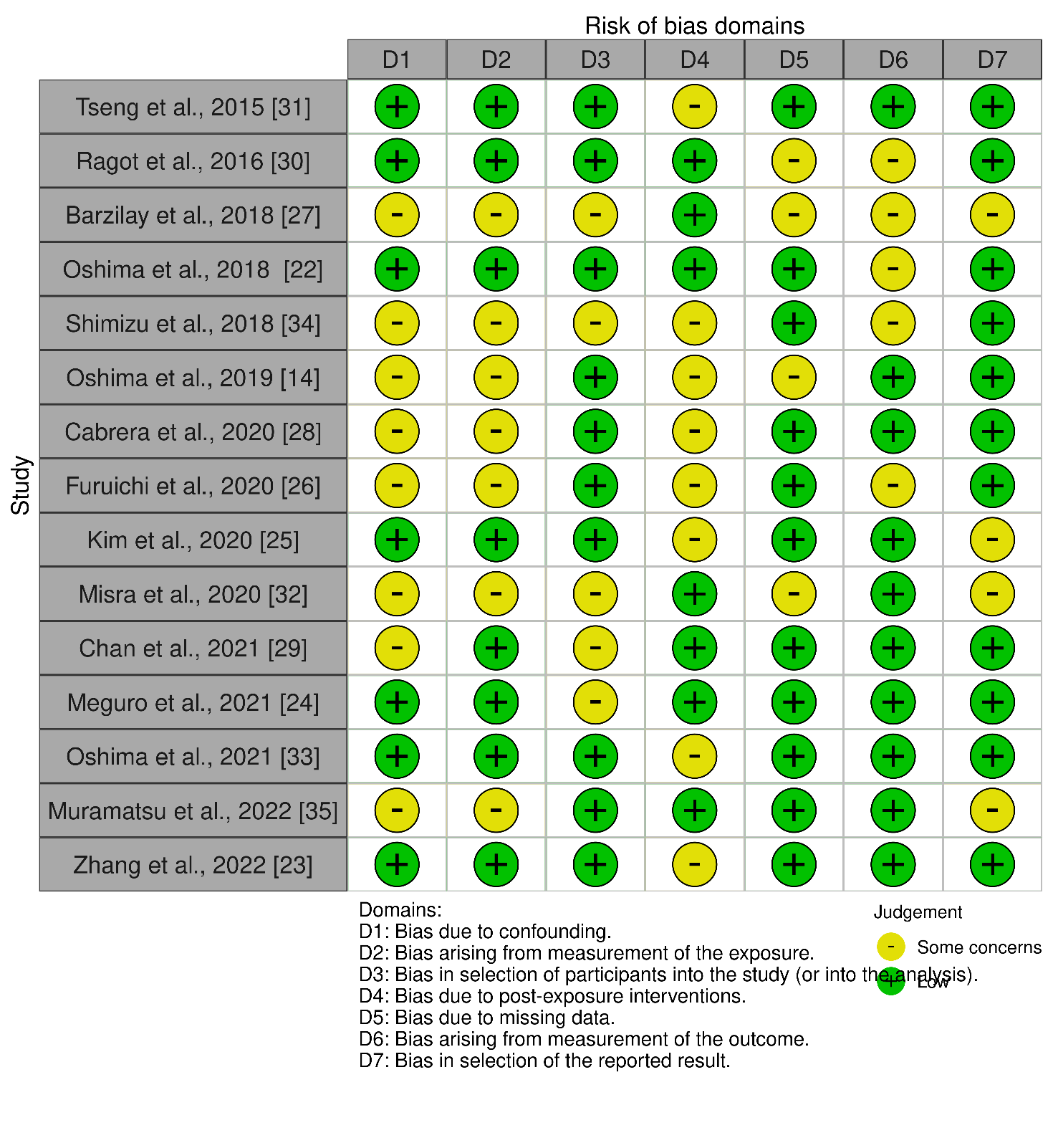


**Figure S1**. Risk of Bias assessment of the studies included in the meta-analysis. According to McGuinness, LA, Higgins, JPT. Risk-of-bias VISualization (robvis): An R package and Shiny web app for visualizing risk-of-bias assessments. *Res Syn Meth.* 2020; 1- 7. https://doi.org/10.1002/jrsm.1411


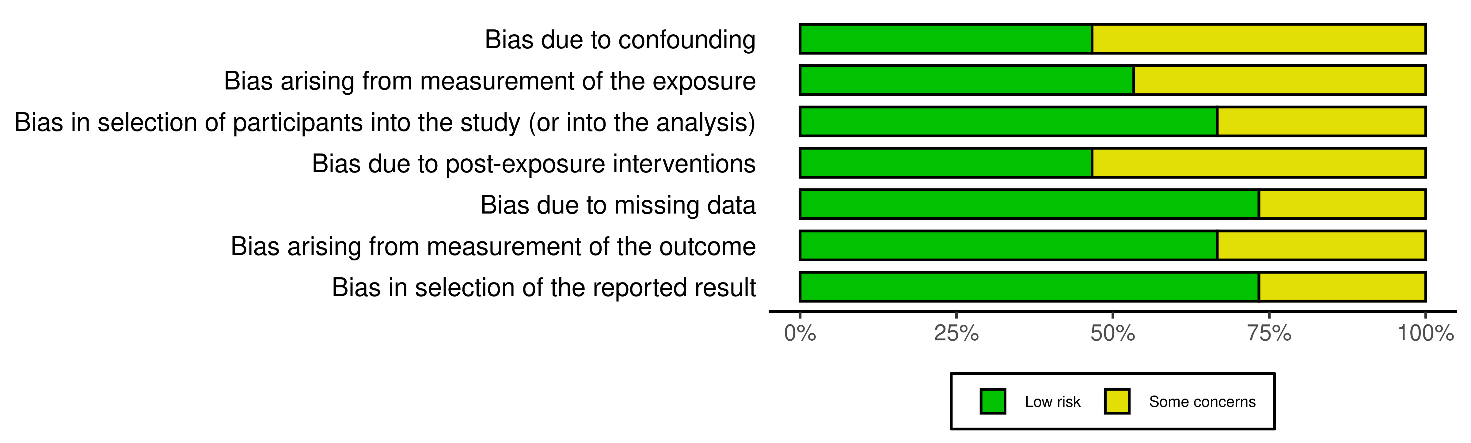


**Figure S2.** Summary Plot of Risk of Bias across the selected papers included in the meta-analysis. According to McGuinness, LA, Higgins, JPT. Risk-of-bias VISualization (robvis): An R package and Shiny web app for visualizing risk-of-bias assessments. *Res Syn Meth.* 2020; 1- 7. https://doi.org/10.1002/jrsm.1411
